# When metabolic prowess is too much of a good thing: how carbon catabolite repression and metabolic versatility impede production of esterified α,ω-diols in *Pseudomonas putida* KT2440

**DOI:** 10.1186/s13068-021-02066-x

**Published:** 2021-11-20

**Authors:** Chunzhe Lu, Christos Batianis, Edward Ofori Akwafo, Rene H. Wijffels, Vitor A. P. Martins dos Santos, Ruud A. Weusthuis

**Affiliations:** 1 Bioprocess Engineering, Wageningen University and Research, Wageningen, The Netherlands; 2 Laboratory of Systems and Synthetic Biology, Wageningen University and Research, Wageningen, The Netherlands; 3 Lifeglimmer GmbH, Berlin, Germany; 4 Faculty of Biosciences and Aquaculture, Nord University, Bodø, Norway

**Keywords:** Metabolic versatility, Substrate preference, Monooxygenase, Medium-chain-length α,ω-diols, ω-Oxyfunctionalization, *Pseudomonas putida*, Esterase

## Abstract

**Background:**

Medium-chain-length α,ω-diols (mcl-diols) are important building blocks in polymer production. Recently, microbial mcl-diol production from alkanes was achieved in *E. coli* (albeit at low rates) using the alkane monooxygenase system AlkBGTL and esterification module Atf1. Owing to its remarkable versatility and conversion capabilities and hence potential for enabling an economically viable process, we assessed whether the industrially robust *P. putida* can be a suitable production organism of mcl-diols.

**Results:**

AlkBGTL and Atf1 were successfully expressed as was shown by oxidation of alkanes to alkanols, and esterification to alkyl acetates. However, the conversion rate was lower than that by *E. coli*, and not fully to diols. The conversion was improved by using citrate instead of glucose as energy source, indicating that carbon catabolite repression plays a role. By overexpressing the activator of AlkBGTL-Atf1, AlkS and deleting *Crc* or *CyoB*, key genes in carbon catabolite repression of *P. putida* increased diacetoxyhexane production by 76% and 65%, respectively. Removing Crc/Hfq attachment sites of mRNAs resulted in the highest diacetoxyhexane production. When the intermediate hexyl acetate was used as substrate, hexanol was detected. This indicated that *P. putida* expressed esterases, hampering accumulation of the corresponding esters and diesters. Sixteen putative esterase genes present in *P. putida* were screened and tested. Among them, Est12/K was proven to be the dominant one. Deletion of *Est12/K* halted hydrolysis of hexyl acetate and diacetoxyhexane. As a result of relieving catabolite repression and preventing the hydrolysis of ester, the optimal strain produced 3.7 mM hexyl acetate from hexane and 6.9 mM 6-hydroxy hexyl acetate and diacetoxyhexane from hexyl acetate, increased by 12.7- and 4.2-fold, respectively, as compared to the starting strain.

**Conclusions:**

This study shows that the metabolic versatility of *P. putida*, and the associated carbon catabolite repression, can hinder production of diols and related esters. Growth on mcl-alcohol and diol esters could be prevented by deleting the dominant esterase. Carbon catabolite repression could be relieved by removing the Crc/Hfq attachment sites. This strategy can be used for efficient expression of other genes regulated by Crc/Hfq in *Pseudomonas* and related species to steer bioconversion processes.

**Supplementary Information:**

The online version contains supplementary material available at 10.1186/s13068-021-02066-x.

## Introduction

### *Pseudomonas putida*: its versatile metabolism and carbon catabolite repression system

*Pseudomonas putida* KT2440 is a well-known Gram-negative bacterium, increasingly attracting industrial attention. Its tolerance to chemical solvents [1], its ability to accumulate large amounts of mcl-PHA [2] and its increasingly available genetic tools [3] make *P. putida* KT2440 a promising chassis for the production of bulk chemicals [4–7]. *P. putida* KT2440 has a versatile metabolism, which is illustrated by its ability to grow on dozens of different carbon sources, including amino acids, alcohols and a wide range of lignin derivatives [8]. In its natural environment, soil, multiple carbon sources are available. To maximize its growth rate, *P. putida* KT2440 needs to select for a preferred substrate. This selection is precisely regulated by carbon catabolite repression (CCR) of essential genes for uptake and catabolism of non-preferred carbon sources [5].

**Fig. 1:**
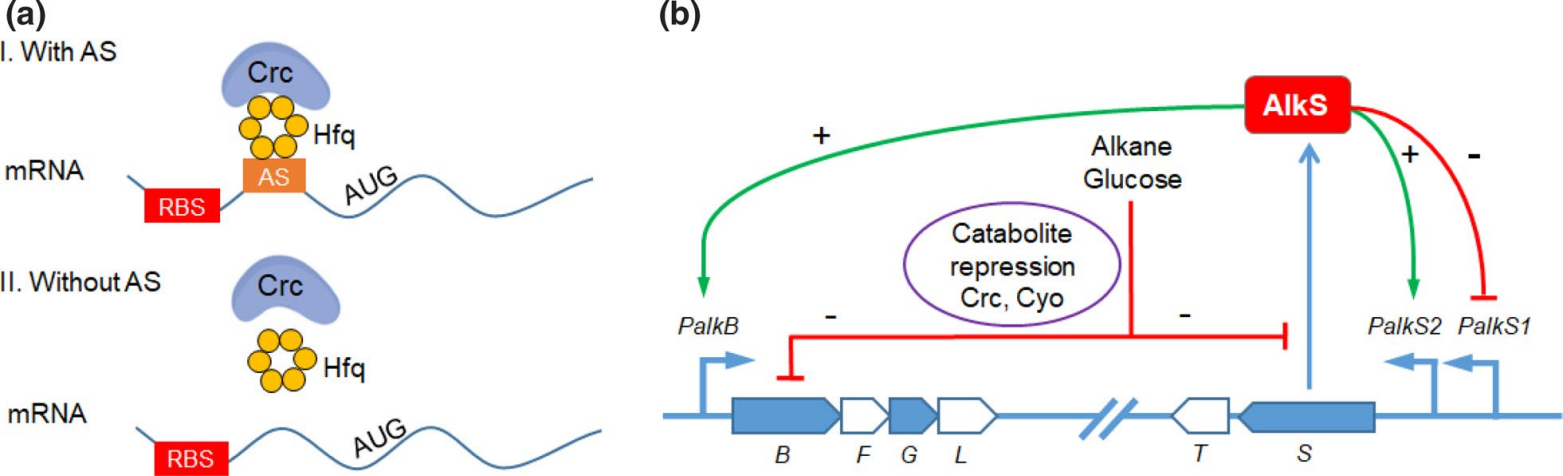
**a** Mechanism of Crc/Hfq binding to attachment sites (AS) on mRNA of target genes. RBS: ribosome binding site. **b** Mechanism of catabolite repression on alkBGTL when glucose and n-alkanes are fed. *AlkS* activator of alkane degradation pathway, *Crc* global regulator of carbon catabolite repression, *Cyo* cytochrome o ubiquinol oxidase, *B* AlkB, alkane monooxygenase, *F* AlkF, rubredoxin which is indispensable for AlkB activity, *G* AlkG, rubredoxin, *L* AlkL, outer membrane protein, *T* AlkT, rubredoxin reductase, *S* AlkS, activator of alkane degradation pathway, *PalkS2* promoter of AlkS with efficient transcription in presence of alkanes, *PalkS1* promoter of AlkS in absence of alkanes, *PalkB* promoter of AlkB

The molecular mechanisms for carbon catabolite repression are primarily controlled by the Crc protein in *Pseudomonas*. Crc regulates the expression of over 130 genes in *P. putida *[9, 10] at the translation level, including those for assimilation of hydrocarbons, such as n-alkanes, benzoate and toluene. Crc forms a complex with the RNA-binding protein Hfq on its distal part in presence of an attachment site (A-rich motifs, AANAANAA) which is located near the translation initiation region of target mRNAs to inhibit translation (Fig. 1a). Except for Crc, the cytochrome o ubiquinol oxidase (Cyo), a terminal oxidase of the electron transport chain, also conduces to catabolite repression. Cyo is considered to play an active role in cell growth under sufficient oxygen supply. Although the mechanism behind Cyo is still unknown, deleting *CyoB*—coding the key gene of Cyo—relieves catabolite repression [11]. Knocking out *Crc* and *CyoB* at the same time showed a superimposed effect on relieving repression in rich medium [11].

These types of CCR systems are necessary for the robustness of microbes in their natural environments when facing multiple substrates, but may impede their performance in industrial biotransformations, in which desired products may act as preferred substrates over the applied substrate[5, 6].

### Microbial synthesis of medium-chain-length α,ω-diols

Medium-chain-length α,ω-diols (mcl-diols) are versatile chemicals used as fuels, detergents and precursors of polyesters and polyurethanes, which are the two primary sources for the production of biobased plastics [12–15]. 1,6-Hexanediol is one of the common α,ω-diols, traditionally produced by hydrogenation of adipic acid [16]. This process requires a high energy input and expensive catalysts, and it usually results in significant byproduct formation. Due to these unsustainable traits, microbial synthesis is attracting more and more attention as a promising alternative solution [17, 18].

Van Nuland and coworkers investigated the conversion of medium-chain alkanes and alkanols into mcl-diols by *E. coli,* by expressing the monooxygenase system AlkBGT from *Pseudomonas putida* GPO1 [19]. In *E. coli* these enzymes act in an orthogonal fashion, as *E. coli* does not employ CCR by the Crc protein. Van Nuland et al. encountered two challenges: AlkBGT overoxidized hydroxyl groups and was unable to oxidize the terminal methyl group of alkanols. They solved the first challenge by protecting the hydroxyl groups by esterifying them with acetyl-CoA using the alcohol acetyltransferase Atf1 from *Saccharomyces cerevisiae.* Glucose was used to provide energy for maintenance and acetyl-CoA for esterification. The esterification of the first hydroxyl group also solved the second challenge as it enabled the ω-oxidation of medium chain alkanol esters. The final products were diacetoxyalkanes: α,ω-diols esterified at both ends with acetate.

In *P. putida* GPO1, the alkBGT system is part of the alkane degradation pathway, encoded on the OCT plasmid [20]*.* AlkB, an integral membrane protein, is an alkane monooxygenase, inserting a single oxygen atom from molecular oxygen into a terminal carbon–hydrogen bond of n-alkanes. The soluble rubredoxin AlkG, which is reduced by the rubredoxin reductase AlkT, is responsible for delivering electrons to AlkB. AlkL is a transporter located in the periplasmic membrane, facilitating uptake of hydrophobic compounds [21, 22]. The alkane degradation genes are split into two clusters, AlkST and AlkBFGHJKL (Fig. 1b). The whole pathway is activated by AlkS controlling the AlkST cluster by the promoter *PalkS2* and the AlkBFGHJKL cluster by the promoter *PalkB*. In *P. putida* GPO1 the Crc protein inhibits translation of AlkS [20], AlkB and AlkG mRNAs to repress AlkBGT activity when cells grow on a minimal medium with preferred substrates such as succinate and lactate^23^, ^24^. *P. putida* KT2440 is relatively close to *P. putida* GPO1, and both share the carbon catabolite repression system mediated by Crc and Cyo [25, 26].

A next challenge in the sustainable microbial production of mcl-diols is to use sugars instead of alkanes as substrate. Owing to its remarkable metabolic versatility, oxidative capacity and industrial robustness, *P. putida* may prove to be a more suitable chassis than *E. coli*, also because of its innate ability to convert sugars into mcl-PHA using the fatty acid synthesis pathway. The intermediates of this pathway can serve as precursors for mcl-diol production. Also the tolerance of some *P. putida* species against chemical solvents such as mcl-diols and their acetate esters may attribute to this suitability [1, 27–29]. On the other hand, expression of AlkBGTL in the presence of glucose is subjected to carbon catabolite repression in *P. putida* and may result in low conversion rates. In addition, another potential issue is that the versatile metabolism of *P. putida* KT2440 possibly enables it to break down the diols and their acetate esters through enzymes like esterases [30].

**Fig. 2:**
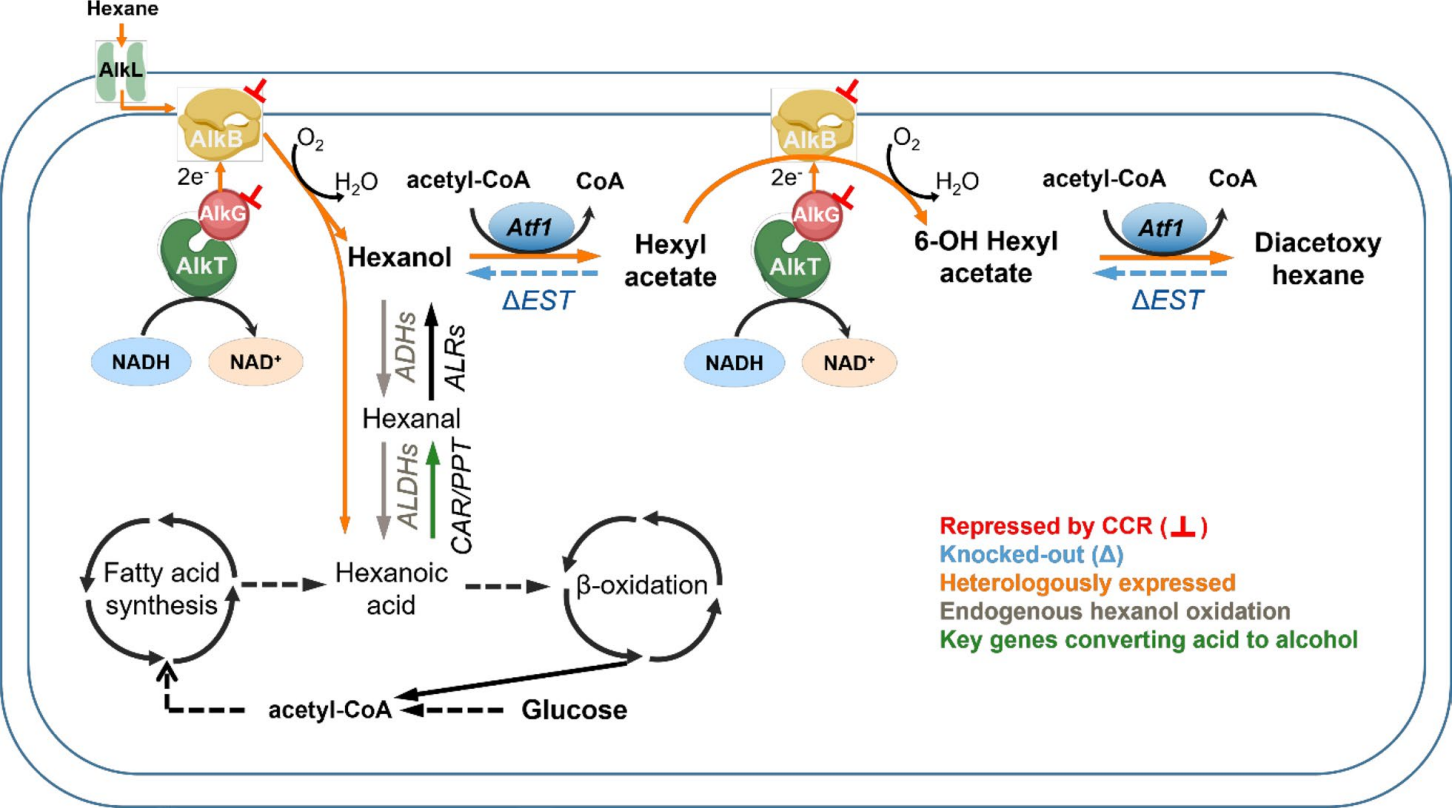
Proposed ω-oxyfunctionalization pathway of medium-chain-length alkyl acetate in *P. putida* KT2440. Orange arrows represent desired pathways, while blue dashed arrows represent breakdown pathways for which esterases are responsible. *CCR* carbon catabolite repression, *AlkB* alkane monooxygenase, *AlkG* rubredoxin, *AlkT* rubredoxin reductase, *Atf1* alcohol acetyl transferase, *EST* esterases, *ADH* alcohol dehydrogenase, *ALDH* aldehyde dehydrogenase, *ALR* aldehyde reductase, *CAR* carboxylic acid reductase, *PPT* phosphopantetheinyl transferase

In this study, *P. putida* KT2440 was engineered as a biocatalyst to produce esterified diols from alkanes by ω-oxidation of alkyl acetate in the presence of glucose (Fig. 2). The monooxygenase system genes *AlkBGTL* and *Atf1* were introduced in *P. putida* KT2440. To ensure efficient function of AlkBGTL, the catabolite repression control gene (*Crc*) and the key gene of cytochrome o ubiquinol oxidase (*CyoB*) were deleted. AlkS as the main activator of the whole oxidation and esterification pathway was overexpressed using the XylS/Pm promoter to counter the potential repression imposed by Crc. Moreover, when the XylS/Pm expression system was used to eliminate carbon catabolite repression, the A-rich motifs of *AlkB*, *AlkG*, and *Atf1*, representing Crc/Hfq attachment sites, were deleted. Alcohol acetyltransferase (Atf1) was expressed to prevent overoxidation and enable terminal oxidation of alcohols. We identified and tested esterases responsible for hydrolysis of alkyl acetate and diol diacetate esters. The dominant esterase was deleted to prevent alkyl acetate from being hydrolysed and to ensure accumulation of esterified diols. This study explores the potential and eliminates hurdles of *P. putida* KT2440 to make it an efficient chassis to further synthesize diols and esters. The strategy can be potentially used for efficient expression of other genes regulated by Crc/Hfq in *Pseudomonas* and related species.

**Fig. 3:**
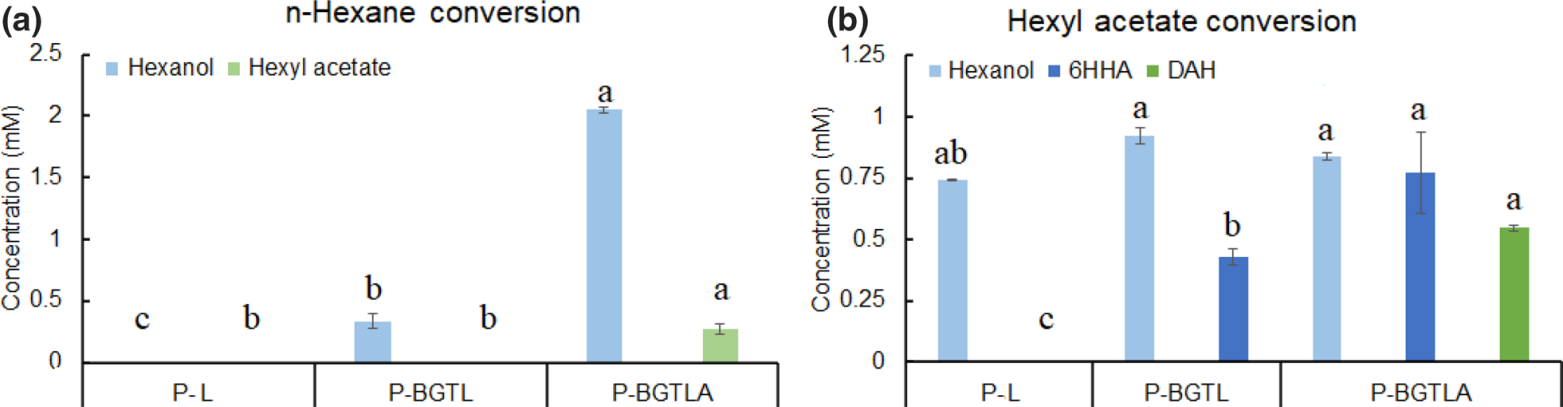
**a** Hexane and **b** hexyl acetate conversion by alkBGTL-Atf1 in *P. putida* KT2440 in 20 h. 1% v/v hexane and hexyl acetate were added as substrates. Glucose was used for cell maintenance and acetyl-CoA supply. *6HHA* 6-hydroxy hexyl acetate, *DAH* 1,6-diacetoxyhexane, *P-L* *P. putida* KT2440 with alkL expression, *P-BGTL* *P. putida* KT2440 with alkBGTL expression, *P-BGTLA* *P. putida* KT2440 with alkBGTL-Atf1 expression. Bars with different letters indicate significant difference from each other (comparisons were implemented among bars with the same color) (p < 0.05)

## Results and discussion

### Introducing AlkBGTL-Atf1 into *P. putida* KT2440

*AlkBGTL* alone and in combination with *Atf1* was introduced into *P. putida* KT2440, resulting in strains P-BGTL and P-BGTLA, respectively. To test the proper functioning of the enzymes, n-hexane and hexyl acetate were separately used as substrates in 1-mL resting-cell suspensions with OD_600_ of 3. When 1% v/v n-hexane (76 mM) was fed in the resting-cell suspensions, P-BGTL produced 0.3 mM 1-hexanol while P-BGTLA produced 2.0 mM 1-hexanol and 0.3 mM hexyl acetate (Fig. 3a) after 20 h. Strain P-L, harboring the substrate transporter alkL, was used as a negative control strain, and did not produce any of these compounds. These results show that both AlkBGTL and Atf1 were successfully expressed in *P. putida*. It was observed that the presence of Atf1 facilitated the production of oxidized products (hexanol and hexyl acetate). P-BGTLA produced far more 1-hexanol than hexyl acetate indicating that either Atf1 is the rate-limiting step or that hexyl acetate produced by Atf1 was hydrolyzed back to 1-hexanol by esterases present in *P. putida* KT2440. Similar experiments have been performed in *E. coli* by van Nuland et al. [19]. When 1% v/v n-hexane was fed to *E. coli* harboring AlkBGTL, over 95% of the total products were overoxidized (hexanoic acid) and minor amounts of 1-hexanol were detected. *E. coli* harboring AlkBGTL-Atf1 produced 9.20 mM 1,6-diacetoxyhexane as the major product and 2.23 mM 6-hydroxy hexyl acetate under same conditions described above. However, hexanoic acid, 6-hydroxy hexyl acetate and 1,6-diacetoxyhexane were not found in our conversions. This may be attributed to a low activity of AlkBGTL in *P. putida* KT2440 resulting from carbon catabolite repression. Similarly, when n-hexane was fed, 1,6-diacetoxyhexane and 6-hydroxy hexyl acetate were not detected in P-BGTLA. We questioned that AlkBGTL-Atf1 could not convert hexyl acetate into 6-hydroxy hexyl acetate and 1,6-diacetoxyhexane in *P. putida*.

To test if hexyl acetate can be converted by AlkBGTL-Atf1, 1% v/v of hexyl acetate (60 mM) was fed to P-L, P-BGTL and P-BGTLA. In a 20-h conversion, 0.77 mM 6-hydroxy hexyl acetate and 0.55 mM 1,6-diacetoxyhexane were detected in P-BGTLA (Fig. 3b). This proves that AlkBGTL-Atf1 is able to ω-oxidize hexyl acetate in *P. putida* KT2440, however, more alcohol products were produced compared to the diester production. 1-Hexanol was found in all strains when hexyl acetate was fed, indicating that esterases were functioning, resulting in the hydrolysis of hexyl acetate to hexanol and acetate.

### Catabolite repression on AlkBGTL-Atf1

**Fig. 4:**
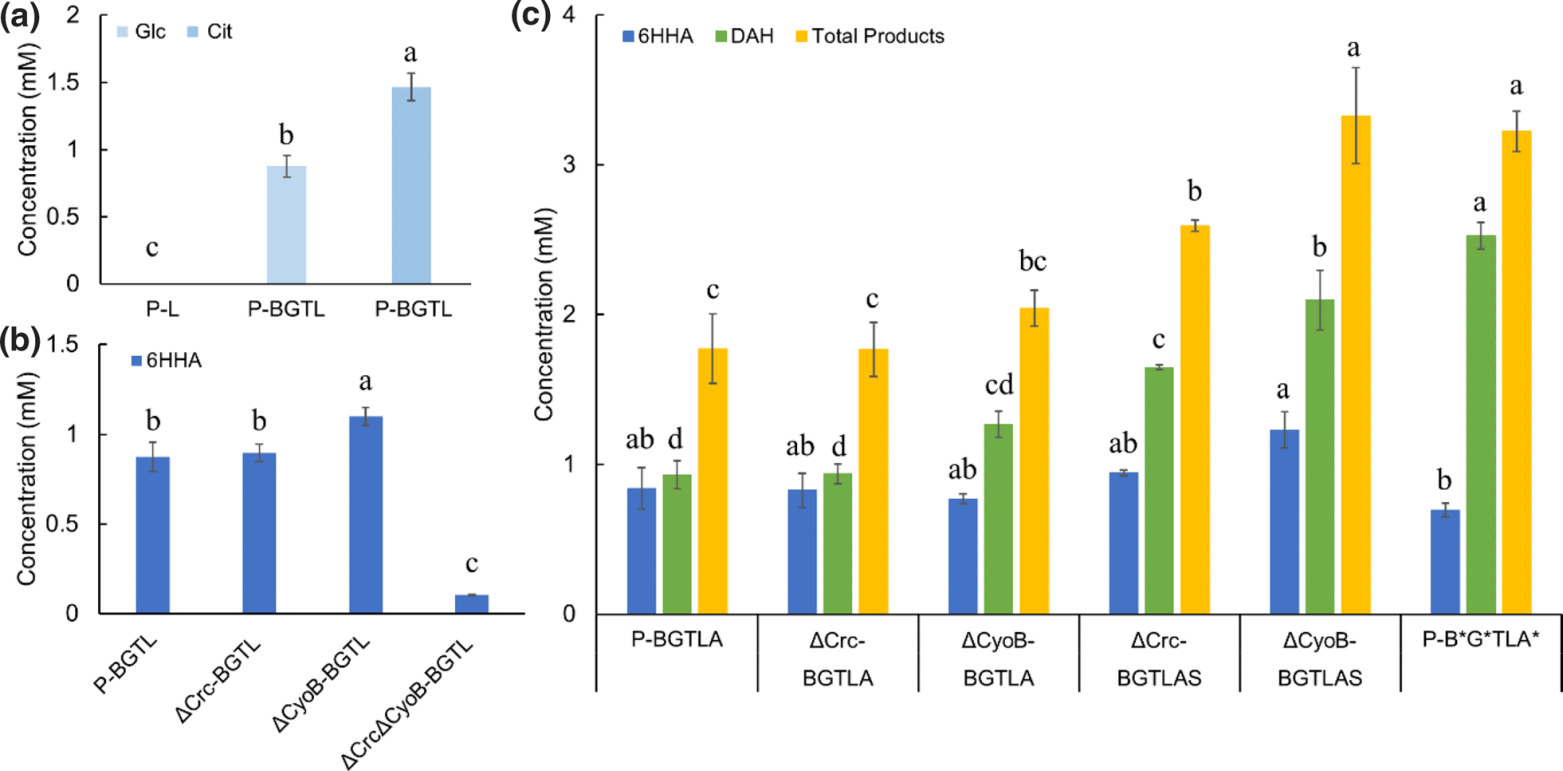
Release of catabolite repression in *P. putida* KT2440. **a** The production of 6-hydroxy hexyl acetate when glucose or citrate was fed to P-BGTL in presence of hexyl acetate. *Glc* glucose, *Cit* citrate. **b** The effect of knockouts of *Crc* and *CyoB* on AlkBGTL when glucose was fed with hexyl acetate. **c** Releasing catabolite repression on AlkBGTL and Atf1 by deleting *Crc* or *CyoB*, overexpressing *AlkS*, and removing the coding sequence of attachment sites of Crc/Hfq when glucose was fed with hexyl acetate. *6HHA* 6-hydroxy hexyl acetate, *DAH* 1,6-diacetoxyhexane, *P-L* *P. putida* KT2440 with alkL expression, *P-BGTL* *P. putida* KT2440 with alkBGTL expression, *ΔCrc-BGTL* *P. putida* KT2440ΔCrc with alkBGTL expression, *ΔCyoB-BGTL* *P. putida* KT2440ΔCyoB with alkBGTL expression, *ΔCrcΔCyoB-BGTL* *P. putida* KT2440ΔCrcΔCyoB with alkBGTL expression, *P-BGTLA* *P. putida* KT2440 with alkBGTL-Atf1 expression, *ΔCrc-BGTLA* *P. putida* KT2440ΔCrc with alkBGTL-Atf1 expression, *ΔCyoB-BGTLA* *P. putida* KT2440ΔCyoB with alkBGTL-Atf1 expression, *ΔCrc-BGTLAS* *P. putida* KT2440ΔCrc with alkBGTL-Atf1 and alkS expression, *ΔCyoB-BGTLAS* *P. putida* KT2440ΔCyoB with alkBGTL-Atf1and alkS expression, P-B*G*TLA* *P. putida* KT2440 with alkBGTL-Atf1 expression, * represents removal of the Crc/Hfq mRNA attachment sites in the genes. Glucose was used for cell maintenance and acetyl-CoA supply for all experiments. Bars with different letters indicate significant difference from each other (comparisons were implemented among bars with the same color) (p < 0.05)

In all experiments, glucose was added to provide energy for maintenance and acetyl-CoA for ester bond formation by Atf1 under resting-cell conditions. n-Hexane or hexyl acetate was fed as substrate to produce hexyl acetate and diacetoxyhexane. However, *P. putida* KT2440 prefers glucose over n-hexane and hexyl acetate, triggering activation of carbon catabolite repression to repress expression and translation of *AlkBGTL *[10]. To test this hypothesis, we tried citrate instead of glucose as it was reported that citrate does not activate carbon catabolite repression of the alkane degradation pathway [10]. Compared to glucose, the production of 6-hydroxy hexyl acetate from hexyl-acetate was increased by 66.9% on citrate reaching 1.5 mM (Fig. 4a). This is in agreement with our hypothesis that glucose represses *AlkBGTL* function in *P. putida* KT2440.

To tackle glucose repression on a molecular level, we used three approaches: (i) knocking out the carbon catabolite repression genes *Crc* and/or *CyoB*; (ii) increasing the expression of the *AlkS* activator; (iii) removing the Crc/Hfq attachment sites from the *AlkBGTL-Atf1* genes.

We deleted *Crc* and *CyoB,* either separately or together, and the mutants were transformed with *AlkL*, *AlkBGTL* and *AlkBGLT-Atf1* to investigate the effects of the regulators on the activity of AlkBGTL, and AlkBGTL-Atf1. It was shown that all mutants, especially the double-knockout strain Δ*Crc*Δ*CyoB*, grew significantly slower than the wild-type strain (results not shown). The results with hexyl acetate as substrate are shown in Fig. 4b when only AlkBGTL was expressed. It shows that the *Crc* knockout had no effect on 6-hydroxy hexyl acetate production and that the *CyoB* knockout increased 6-hydroxy hexyl acetate by 25.6%. When both *Crc* and *CyoB* were deleted, 6-hydroxy hexyl acetate production reduced with 88.0%. This conflicts with previous research performed by Dinamarca and his colleagues [11] who showed that Crc and Cyo have an additive influence on the alkane degradation pathway. However, they used the *lacZ* gene as an orthogonal reporter to replace alkane degradation genes to test how knockouts affect gene expression under the control of the promoter *PalkB*. In our case, AlkBGTL was directly used to produce 6-hydroxy hexyl acetate, and was connected to the rest of metabolism, e.g. due to NADH requirement. Because deletion of *Crc* and *CyoB* will affect the expression of many genes [31], the whole metabolism will be significantly changed, which may have led to poor availability of NADH. This may be the reason why strain ΔCrcΔCyoB-BGTL only produced trace amounts of 6-hydroxy hexyl acetate.

To test the performance of AlkBGTL-Atf1 in the knockouts strains, the plasmid pBGTL-Atf1 was separately introduced, generating strains P-BGTLA, ΔCrc-BGTLA, and ΔCyoB-BGTLA. Compared to P-BGTLA, ΔCrc-BGTLA produced the same amount of 6-hydroxy hexyl acetate and diacetoxyhexane (Fig. 4c), indicating that the *Crc* knockout cannot increase AlkBGTL-Atf1 activity. The diacetoxyhexane production by strain ΔCyoB-BGTLA increased by 36.5% in comparison with the P-BGTLA strain, indicating that esterification was improved. The knockout of *CyoB* might release the repression on *Atf1* or limit electron transport chain to provide more acetyl-CoA to Atf1. In AlkBGTL-Atf1 case, not only *AlkS*, *AlkB*, and *AlkG*, but also *Atf1* were repressed because *Atf1* was controlled by PalkB too. However, because Crc represses the alkane degradation pathway mainly through reducing the expression level of *AlkS*, which is the activator of the whole pathway, AlkS was reportedly overexpressed to counter repression of Crc on the pathway [24]. In this work, pSEVAb628-AlkS was, respectively, transformed into strains ΔCrc-BGTLA and ΔCyoB-BGTLA, giving rise to strains ΔCrc-BGTLAS and ΔCyoB-BGTLAS to overexpress AlkS. Strain ΔCrc-BGTLAS produced 14% more 6-hydroxy hexyl acetate and 76% more diacetoxyhexane, while strain ΔCyoB-BGTLAS produced 60% more 6-hydroxy hexyl acetate and 65% more diacetoxyhexane (Fig. 4c). But, because Crc and Cyo are global regulators, knocking them out affects the whole metabolism of *P. putida*, as indicated by the reduced growth rates.

**Fig. 5:**
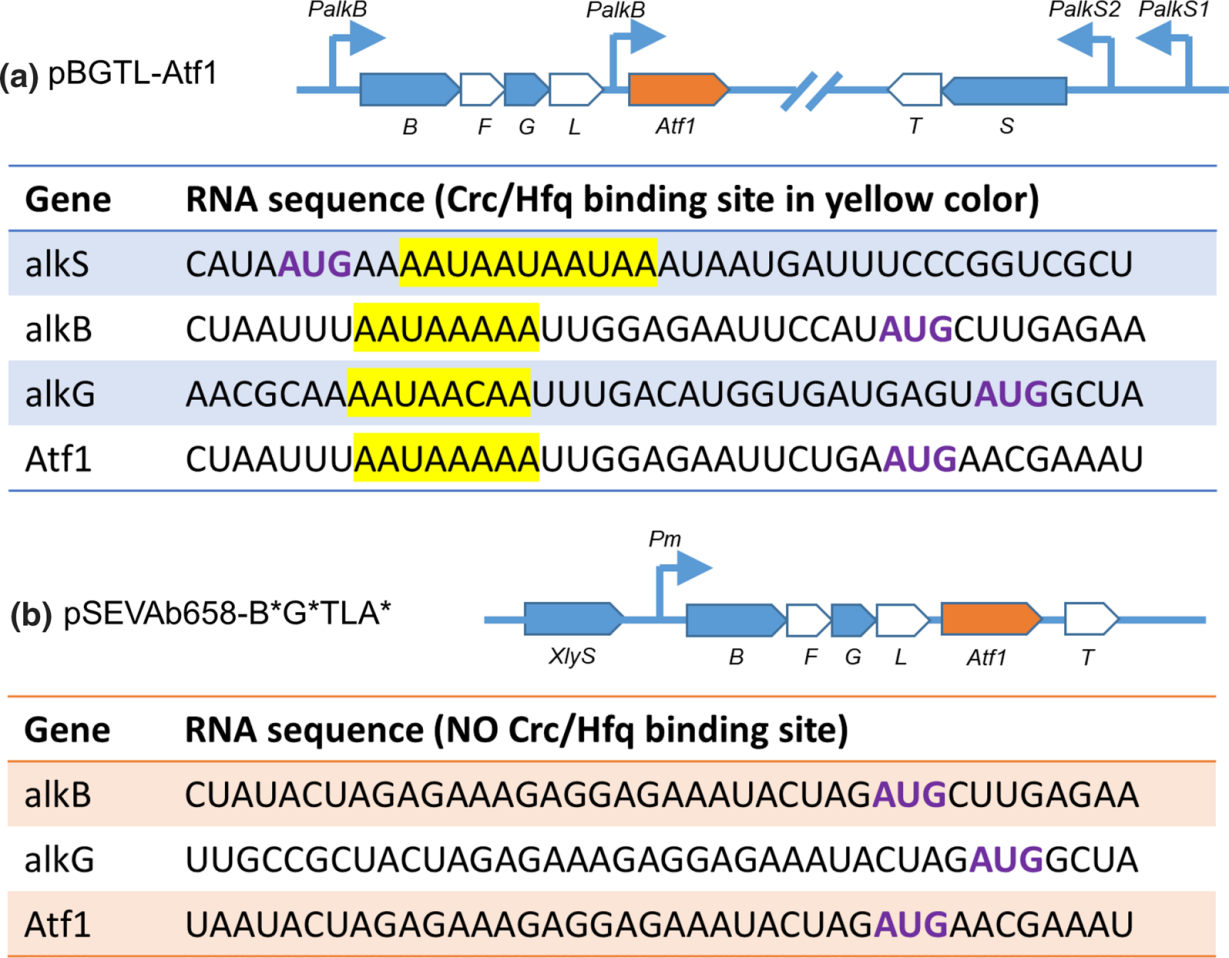
The schematic structure of plasmids (a) pBGTL-Atf1 and (b) pSEVAb658-B^*^G^*^TL-Atf1^*^. * represents removal of the Crc/Hfq mRNA attachment sites in the genes

To establish less pleiotropy, we decided to remove the sequences encoding the mRNA attachment sites of the Crc/Hfq complex from the overexpressed genes. The attachment sites in front of *AlkB*, *AlkG* and *Atf1* were removed, generating a plasmid pSEVAb658-alkB^*^G^*^TL-Atf1^*^(Fig. 5), which was subsequently transformed into *P. putida* KT2440 resulting in strain P-B^*^G^*^TLA^*^. Compared with P-BGTLA, the total production of P-B^*^G^*^TLA^*^ was increased by 82%. Among all five tested strains (ΔCrc-BGTLA, ΔCyoB-BGTLA, ΔCrc-BGTLAS, ΔCyoB-BGTLAS, and P-B^*^G^*^TLA^*^), ΔCyoB-BGTLAS and P-B^*^G^*^TLA^*^ were the two best-performing strains, producing 3.3 and 3.2 mM total products (6-hydroxy hexyl acetate and diacetoxyhexane), respectively (Fig. 4c). The difference between ΔCyoB-BGTLAS and P-B^*^G^*^TLA^*^ was the ratio of 6-hydroxy hexyl acetate and diacetoxyhexane. P-B^*^G^*^TLA^*^ showed the highest diacetoxyhexane concentration (2.52 mM) while ΔCyoB-BGTLAS generated the highest 6-hydroxy hexyl acetate concentration (1.2 mM). AlkS was overexpressed in ΔCyoB-BGTLAS to ensure high expression of AlkBGTL-Atf1 to counter carbon catabolite repression, leading to the highest total products. But 6-hydroxy hexyl acetate is not fully converted to diacetoxyhexane, indicating esterification of 6HHA is still limited. P-B^*^G^*^TLA^*^ significantly released the inhibition of Atf1, facilitating higher production of diacetoxyhexane. The plasmid pSEVA658-B^*^G^*^TL-Atf1^*^ was used for further tests, given its high production and lower pleiotropy.

### Growth test of *P. putida* KT2440 on hexyl acetate and DAH

*P. putida* KT2440, known for its versatile metabolism, can grow on dozens of different carbon sources [5–8]. To date, it is unknown whether *P. putida* KT2440 can utilize aliphatic acetate esters and diesters for growth. In our study, hexyl acetate was hydrolyzed to hexanol (Fig. 3b), which can be easily further utilized via β-oxidation. Thus, we assumed *P. putida* KT2440 is able to use hexyl acetate and diacetoxyhexane as the only carbon source for growth, in which esterases perform the first step, the hydrolysis of esters. If so, this is a potential threat for *P. putida* KT2440 to be engineered for hexyl acetate and diacetoxyhexane production.

**Fig. 6:**
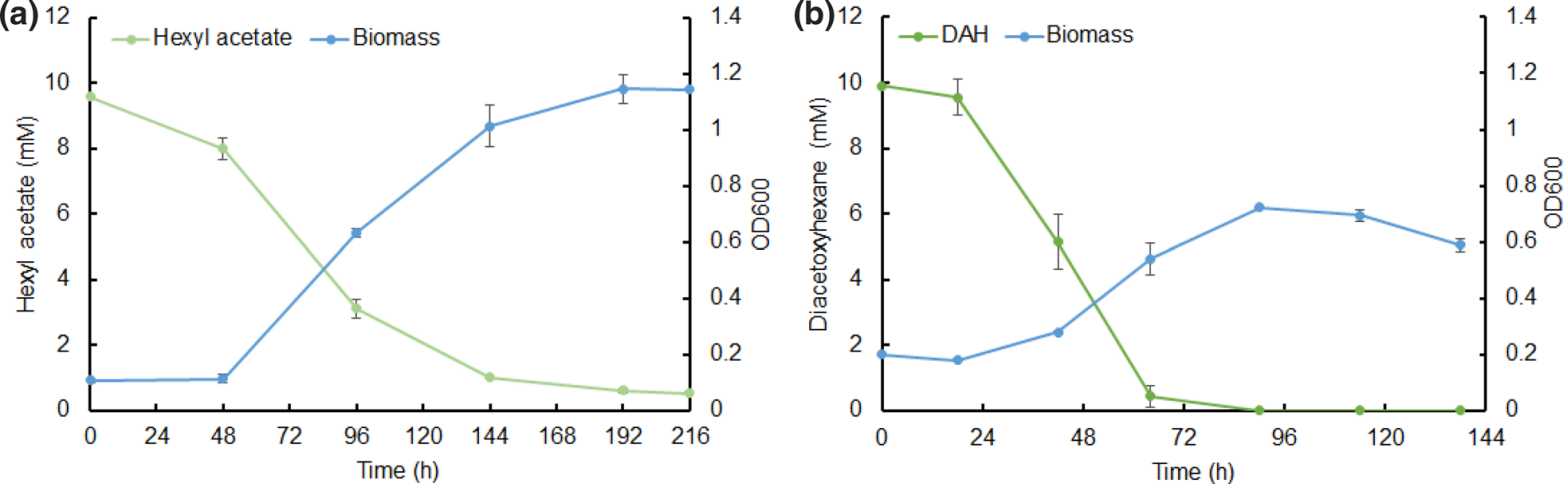
Growth curves of *P. putida* KT2440 on 10 mM **a** hexyl acetate and **b** diacetoxyhexane (DAH). Hexyl acetate or diacetoxyhexane was used as the only carbon source for cell growth. Each growth experiment was performed in triplicate

Wild-type *P. putida* KT2440 was used for growth tests on M9 medium with 10 mM hexyl acetate or diacetoxyhexane as the only carbon source. The hexyl acetate case (Fig. 6a) showed a 48 h lag phase, after which the biomass concentration increased quickly and the hexyl acetate concentration decreased. In the end, 95% of the initially added hexyl acetate was consumed and the optical density reached its maximum at 1.15 with an OD_600_ increase of 1.05. In the diacetoxyhexane case, the biomass concentration increased and the diacetoxyhexane concentration reduced over time (Fig. 6b). After a 24-h lag phase, the diacetoxyhexane concentration rapidly decreased until it was completely consumed at 72 h. Nevertheless, the maximum optical density was only 0.72 with an OD_600_ increase of 0.52, half of the OD increase of the hexyl acetate case. The difference between these two cases is that 1-hexanol was hydrolyzed from hexyl acetate and 1,6-hexanediol was obtained from diacetoxyhexane breakdown. According to Li et al. [30], 1,6-hexanediol cannot be utilized by the native metabolism of *P. putida*, while 1-hexanol can be easily assimilated. So in the diacetoxyhexane case, only the released acetate could be used as carbon source for cell growth. Therefore, a higher biomass concentration was obtained when hexyl acetate was fed. In summary, *P. putida* KT2440 is able to use hexyl acetate or diacetoxyhexane as the only carbon source to grow. We hypothesized that hydrolysis of hexyl acetate into 1-hexanol and acetate or hydrolysis of diacetoxyhexane into 1,6-hexanediol and acetate is the first and essential step for cell growth. Esterases are supposed to be responsible for this hydrolysis [32–34]. To prevent their action, we verified the presence and activity of relevant esterases.

### Screening of esterases in *P. putida* KT2440

In *P. putida* KT2440, several esterase genes have been reported, such as *EstZ* and *EstA *[35]. However, it is unclear how many esterases were active in the above cases. To check for potential esterases, searches were performed based on the genome of *P. putida* KT2440 using the NCBI and CAZymes databases. As a result, 16 putative esterases were selected (Table 2).

To evaluate the performance of these 16 esterases, *E. coli* NEBT7 was used as a host for quick screening due to its relatively clear esterase background compared with *P. putida* KT2440. *E. coli* NEBT7 transformed with an empty pET26b plasmid was used as control strain. pET26b plasmids harboring the (putative) esterase genes were introduced into *E. coli* NEBT7. The transformed strains were used for monoester and diester hydrolysis.

**Fig. 7:**
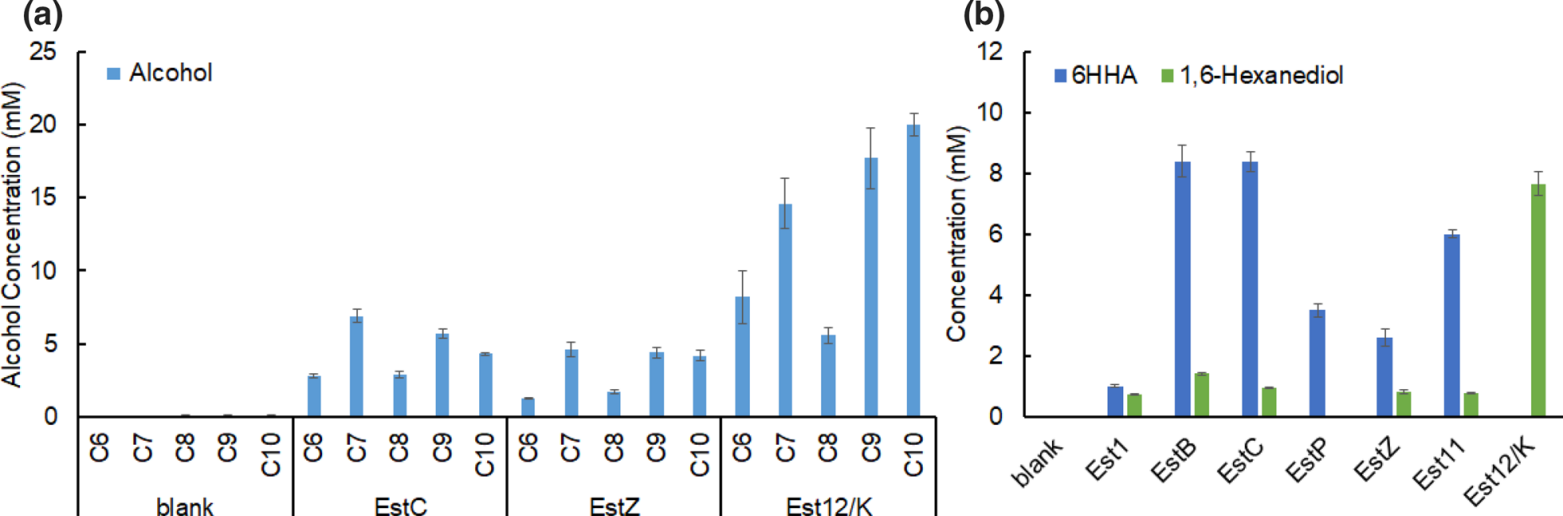
Alkyl (C6–C10) acetate ester (**a**) and diacetoxyhexane (**b**) hydrolysis test by resting-cell of *E. coli*-pET26b-*EstX* in 20 h. EstX represents each putative esterase gene. Glucose was used to provide energy for cell maintenance. 6HHA: 6-hydroxy hexyl acetate

Induced resting *E. coli* cells (1 g_cdw_/L) were fed with 20 mM alkyl (C6-C10) acetate ester or diacetoxyhexane in tightly capped tubes. Production of mcl-n-alcohols (C6–C10), 6-hydroxy hexyl acetate and 1,6-hexanediol was used as an indicator to confirm esterase activity. According to these criteria, three esterases (EstC, EstZ, Est12/K) were active in alkyl acetate ester hydrolysis (Fig. 7a). The rest of esterases showed no alcohol production (results not shown). Est12/K was always the best-performing esterase, no matter which alkyl acetate ester was fed. Of the 16 candidates seven esterases—Est1, EstB, EstC, EstP, EstZ, Est11, Est12/K—showed diacetoxyhexane hydrolysis activity, either to 6-hydroxy hexyl acetate or 1,6-hexanediol (Fig. 7b). Among these seven esterases, Est12/K showed the best hydrolysis performance producing 7.7 mM 1,6-hexanediol, followed by EstB, EstC and Est11 exhibiting almost the same ability to hydrolyze diacetoxyhexane. Est1 only produced trace amounts of 6-hydroxy hexyl acetate and 1,6-hexanediol.

### Evaluation of the deletion of esterase in *P. putida* KT2440

In the above analysis, esterases were highly expressed in *E. coli*. However, they might show different activity in *P. putida* KT2440 due to different expression levels of the esterase and diverse metabolism. To investigate their performance in *P. putida*, esterase knockout (KO) mutants were generated. Single KO and multiple KO mutants of *EstB*, *EstC*, *EstP*, *EstZ* and *Est12/K* in *P. putida* KT2440 were made and evaluated in hydrolysis tests. In addition to esterases, β-oxidation was assumed to be involved in consumption of alcohols from ester hydrolysis, facilitating this hydrolysis. Thus, key genes (*FadA*: acetyl-CoA acetyltransferase (PP_2137, PP_2215), *FadB*: 3-hydroxyacyl-CoA dehydrogenase/enoyl-CoA hydratase (PP_2047, PP_2136, PP_2214, PP_2217), *FadE*: acyl-CoA dehydrogenase (PP_2048, PP_2216) [36, 37] of β-oxidation were deleted, generating *P. putida* ΔBOX as a control strain.

**Fig. 8:**
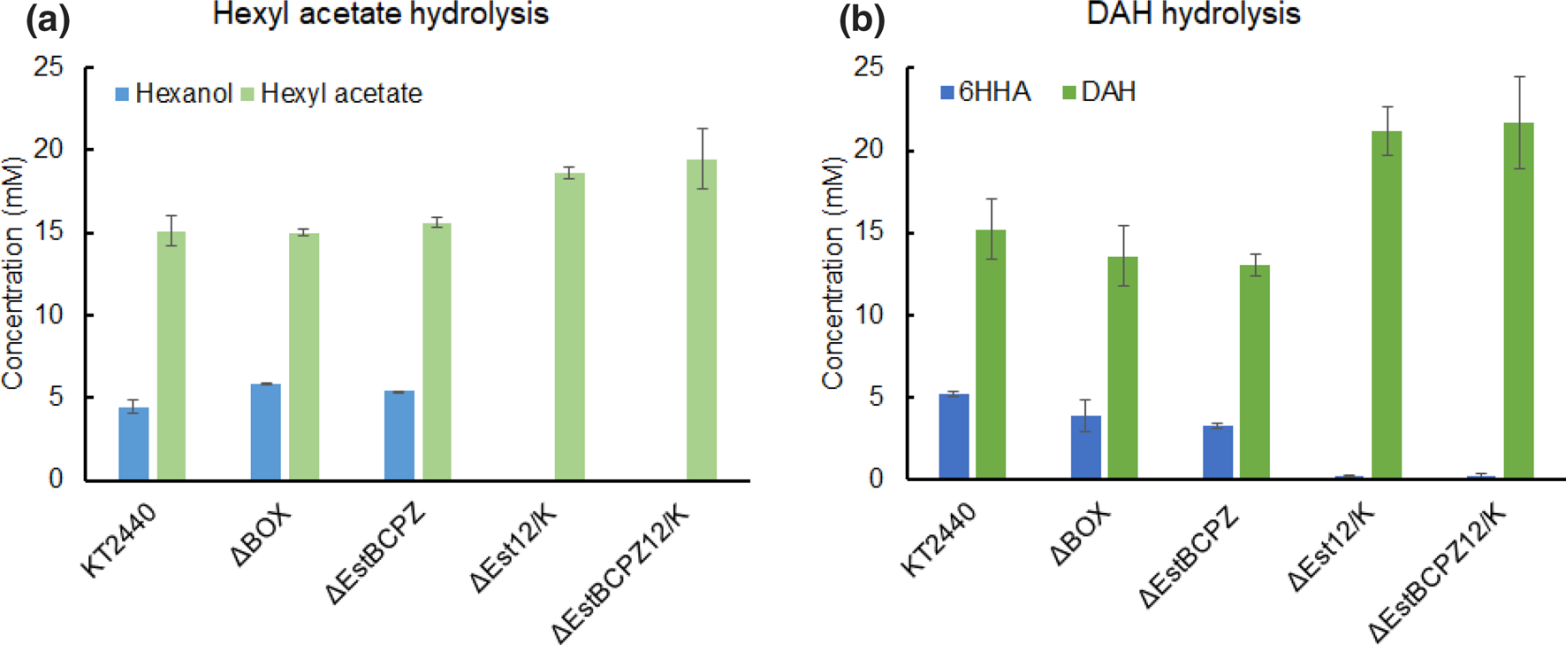
Esterase deletion mutants of *P. putida* KT2440 for **a** 20 mM hexyl acetate hydrolysis test in 48 h and **b** 20 mM diacetoxyhexane hydrolysis test in 24 h. Glucose was used for cell maintenance. *6HHA* 6-hydroxy hexyl acetate, *DAH* 1,6-diacetoxyhexane, *ΔBOX* *P. putida* KT2440ΔPP_2014-18ΔPP_2047-48ΔPP_2136-37, *ΔEstBCPZ* *P. putida* KT2440ΔEstB ΔEstCΔEstPΔEstZ, *ΔEst12/K* *P. putida* KT2440ΔEst12/K, *ΔEstBCPZ12/K* *P. putida* KT2440ΔEstBΔEstCΔEstPΔEstZΔEst12/K

For alkyl acetate ester hydrolysis in *P. putida* and esterase KO mutants, hexyl acetate (20 mM) was taken as example. The control strains, *P. putida* KT2440 and *P. putida* ΔBOX, formed around 5.5 mM 1-hexanol after 48 h of incubation (Fig. 8a). This shows that under the incubation conditions applied, β-oxidation did not play a significant role in alcohol consumption. The ΔEstBCPZ strain showed similar results, indicating that EstB, EstC, EstP and EstZ are not involved in hexyl acetate hydrolysis in *P. putida* cultivated under these conditions. When *Est12/K* was deleted, 1-hexanol was not detected anymore. For diacetoxyhexane hydrolysis, the data showed a similar trend (Fig. 8b). The intermediate ester, 6-hydroxy hexyl acetate, the product of a single hydrolysis, was not observed. In brief, Est12/K is the most dominant esterase responsible for the breakdown of alkyl acetate and diacetoxyhexane in *P. putida* KT2440.

### Diacetoxyhexane production in engineered *P. putida* KT2440

**Fig. 9:**
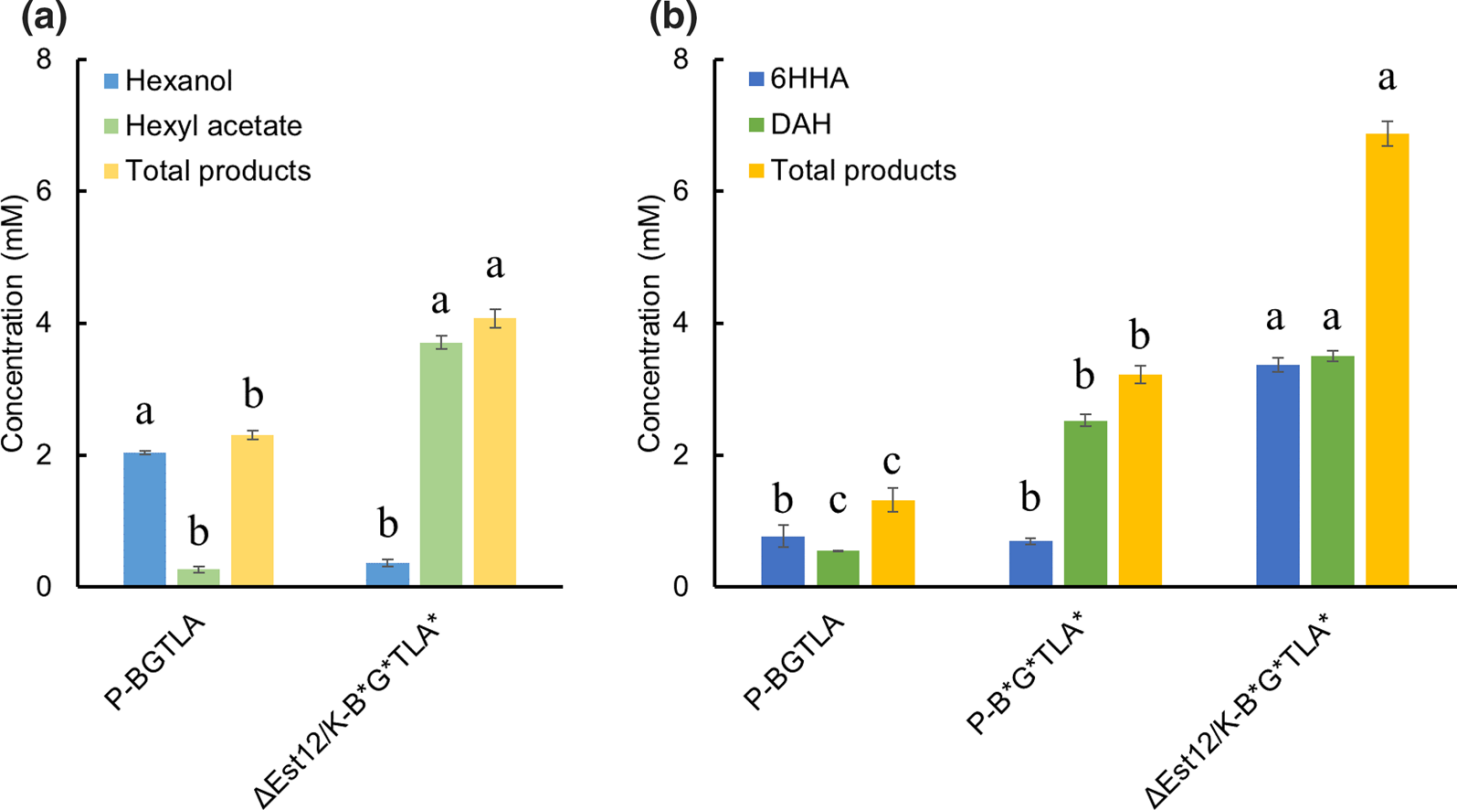
Oxidation conversion of n-hexane and hexyl acetate by engineered strains under resting-cell conditions. **a** Hexyl acetate production from 1% v/v n-hexane in engineered *P. putida* KT2440. **b** DAH production from 1% v/v hexyl acetate in engineered *P. putida* KT2440. Glucose was fed for cell maintenance. *6HHA* 6-hydroxy hexyl acetate, *DAH* 1,6-diacetoxyhexane, *P-BGTLA* *P. putida* KT2440 with alkBGTL-Atf1 expression, P-B^*^G^*^TLA^*^ *P. putida* KT2440 with expression of alkBGTL-Atf1 where the Crc/Hfq mRNA attachment sites of alkB, alkG and Atf1 were removed, ΔEst12/K-B^*^G^*^TLA^*^ *P. putida* KT2440 ΔEst12/K expression of alkBGTL-Atf1 where the Crc/Hfq mRNA attachment sites of alkB, alkG and Atf1 were removed. Bars with different letters indicate significant difference from each other (comparisons were implemented among bars with the same color) (p < 0.05)

In the next step, we combined the esterase knockout strain with the expression of Crc-independent AlkBGTL-Atf1. pSEVA658-B^*^G^*^TL-Atf1^*^ was transferred to *P. putida* ΔEst12/K giving rise to ΔEst12/K-B^*^G^*^TLA^*^. When n-hexane was used as substrate in resting-cell suspension of strain ΔEst12/K-B^*^G^*^TLA^*^, 0.4 mM hexanol and 3.7 mM hexyl acetate were produced. The production of hexyl acetate was increased by 12.7-fold compared with that of P-BGTLA. This mainly resulted from the deletion of *Est12/K*, preventing the hydrolysis of hexyl acetate. Besides, optimized AlkBGTL also provides more hexanol for hexyl acetate formation. However, 6-hydroxy hexyl acetate and 1,6-diacetoxyhexane were still not detected (data not shown). In a similar setup, using hexyl acetate as substrate, ΔEst12/K-B^*^G^*^TLA^*^ produced 6.9 mM total products (Fig. 9b). Compared with P-B^*^G^*^TLA^*^, ΔEst12/K-B^*^G^*^TLA^*^ produced 1.1-fold more total products. The production of 6-hydroxy hexyl acetate increased by 3.8-fold, indicating that 6-hydroxy hexyl acetate might be hydrolyzed when Est12/K is present. In the end, the total amount of products was increased by 4.2-fold in ΔEst12/K-B^*^G^*^TLA^*^ compared with the starting strain P-BGTLA. 11.5% of the substrate (hexyl acetate) was ω-oxidized by ΔEst12/K-B^*^G^*^TLA^*^, 4.2-fold of that of P-BGTLA.

Both interventions, preventing hydrolysis of esters and uncoupling ester production from carbon catabolite repression, resulted in improved ester production. However, ester production is still lower in *P. putida* compared with that in *E. coli*. There are three main differences between the two hosts for ester synthesis. First, in a similar setup *E. coli* showed a threefold higher production rate when hexane was used as starting substrate [19]. The lower production rate of *P. putida* may be attributed to the availability of NADH—required for AlkBGT activity—and acetyl-CoA—required for Atf1 activity. Whereas *E. coli*’s dissimilation is based on NADH, *P. putida*’s dissimilation has a stronger focus on NADP^+^ reduction, mediated by the ED-EMP pathway [38]. Although this is generally an advantage in *P. putida* with regard to its metabolic prowess, in this specific case, this may have resulted in a low availability of NADH and hence a lower AlkBGT activity. Glucose was added to provide the acetyl-CoA. However, when glucose was fed to *P. putida*, the majority of glucose is not directly assimilated but is channelled to periplasmic gluconate and 2-ketogluconate pathways, and secreted in the presence of abundant glucose [39], limiting the supply of acetyl-CoA. In addition, glucose is the preferred substrate for *E. coli*, but not for *P. putida *[10]. The conversion of hexane results in the accumulation and excretion of many intermediate molecules. Some of these molecules may be preferred as substrate over glucose, resulting in a lower availability of acetyl-CoA and hence a lower Atf1 activity. The same reasoning, based on the difference in substrate preference between *E. coli* and *P. putida* may also be applied to the second difference found. In *E. coli* AlkBGT overoxidizes a part of the hexanol into hexanoic acid. Esterification of hexanol and 6-hydroxy hexyl acetate with acetyl-CoA mediated by Atf1 was not able to prevent this completely. None of hexanoic acid, 6-hydroxyhexanoate, and adipic acid were observed as byproduct using *P. putida*. This can however not be attributed to a high activity of Atf1, because both hexanol and 6-hydroxy hexyl acetate accumulated. It seems more likely that overoxidation did occur, but that the overoxidized product hexanoic acid was possibly preferred as substrate over glucose and hexane. The third difference is that in *E. coli* hexane can be directly converted into diacetoxyhexane. This direct conversion does not occur in *P. putida*, although it is able to convert hexane into hexyl acetate and added hexyl acetate in diacetoxyhexane. The ability of AlkBGT to oxidize both hexane and hexyl acetate simultaneously in *E. coli* and not in *P. putida* suggests that in the latter microorganism the ratio of intracellular concentrations of hexane and hexyl acetate is larger, favoring hexane oxidation by substrate competition.

## Conclusions

*P. putida* KT2440 is known for its robustness, versatile metabolism and high oxidative capacity, all of which render it a preferred platform for a range of industrial applications [6–8, 40, 41]. This study shows that this metabolic prowess also has its down side. We found that metabolic versatility and associated carbon catabolite repression hinder oxyfunctionalization of alkanes by alkBGTL-Atf1 in *P. putida* KT2440. Both could be overcome by 1) deleting the committed step of diacetoxy alkane degradation performed by esterase Est12/K and 2) by deleting the Crc/Hfq attachment site of relevant genes involved in oxyfunctionalization to avoid catabolite repression. Nevertheless, the realized level of diacetoxy alkane production is still low, and more research is required to make the process industrially applicable.

Although the role of catabolite repression in direct product formation was diminished, the results also indicate that the formed (by)products may still affect other parts of the metabolic network by carbon catabolite repression. Biotechnological process development is largely about engineering the most (cost-)effective conversion of a chosen substrate into a chosen product. The substrate preference of *P. putida* may conflict with these choices, if the product is preferred as substrate. This phenomenon is already known as the Kluyver effect for some yeasts, which are not able to convert some disaccharides into ethanol, because ethanol is preferred as substrate [42]. Sugars are often the main substrates used in biotechnological processes. Sugars are, however, not the preferred substrates of *P. putida*. This ultimately hampers the ability to use *P. putida* for the production of certain compounds, e.g. organic acids and amino acids. The strategy we used to delete mRNA Crc/Hfq attachment sites may alleviate this when applied to genes involved in sugar catabolism for other applications. Moreover, the ability to regulate many enzyme activities at the same time with Crc/Hfq, as long as the genes involved contain an (introduced) mRNA attachment site, could be a promising tool in metabolic engineering.

## Materials and methods

### Strains, plasmids and medium

Strains and plasmids used in this study are listed in Table 1. *E. coli* 5α was used for cloning purposes. *E. coli* NEBT7 was used for ester hydrolysis tests. The construction of plasmids is described in the supplementary file. *P. putida* KT2440 as the parental strain was engineered for different study aims. According to Nicolas and Daniel’s method [43], a suicide plasmid pGNW2 and a self-curing plasmid pQURE6⋅H bearing the I-SceI gene were used to delete genes in *P. putida* KT2440. The M9 medium consisted of 1 × M9 minimal salts, 0.2 mM MgSO_4_·7H_2_O, 55 mM glucose and 1 mL/L trace elements US^Fe^ [44]. M9^E^ medium was used for cell preculture and induction of genes of interest. It consisted of M9 medium, 0.1 mM CaCl_2_·2H_2_O, 100 mM 3-(N-morpholino) propanesulfonic acid sodium salt buffer (MOPS), and 1 mL/L 1000 × vitamins [45]. The media were adjusted to pH 7.0 with 1 M hydrochloric acid or sodium hydroxide and subsequently filter-sterilized using a 0.22 µm Nalgene® polyethersulfone (PES) filter (ThermoFisher). Kanamycin (Km) and gentamicin (Gm) were separately added at 50 μg/mL and 10 μg/mL, respectively, when needed.

**Table 1:** Plasmids and strains used in this work

Name	Description	References
Plasmids		
pCOM_*AlkL*	pCOM10 vector containing *AlkL* gene; Km^R^	[22]
pBTL10	pCOM10 vector containing *AlkBGTL* genes; Km^R^	[50]
pBGTL-Atf1	pCOM10 vector containing *AlkBGTL-Atf1* genes; Km^R^	[19]
pSEVAb628	Expression vector; *oriV(RK2)*; *XylS, Pm*; Gm^R^	[47]
pSEVAb658	Expression vector; *oriV(RSF1010)*; *XylS, Pm*; Gm^R^	[47]
pSEVAb628-AlkS	pSEVAb628 vector containing *Pm → AlkS*; Gm^R^	This work
pSEVAb658-B^*^G^*^TLA^*^	pSEVAb658 vector containing Pm → AlkB^*^G^*^TL-Atf1^*^; Gm^R^	This work
pET26b-EstX	pET26b vector containing *T7 → EstX*, X represents each putative esterase gene; Km^R^	This work
pGNW2	Suicide vector used for gene deletion in *P. putida* KT2440; *oriV(R6K)* containing *P14g → msfGFP*; Km^R^	[43]
pGNW2-ΔCrc	Derivative of vector pGNW2 containing HRs to delete *Crc* (*PP_5292*)	This work
pGNW2-ΔCyoB	Derivative of vector pGNW2 containing HRs to delete *CyoB* (*PP_0813*)	This work
pGNW2-ΔEstX	Derivative of vector pGNW2 containing HRs to delete *EsteraseX* (Table 2)	This work
pGNW2-PP_2047-48	Derivative of vector pGNW2 containing HRs to delete *PP_2047-48*	This work
pGNW2-PP_2136-37	Derivative of vector pGNW2 containing HRs to delete *PP_2136-37*	This work
pGNW2-PP_2214-18	Derivative of vector pGNW2 containing HRs to delete *PP_2214-18*	This work
Strains		
* P. putida* KT2440	Wild-type strain, derived from *P. putida* mt-2[51]	[52]
ΔCrc	*P. putida* KT2440Δ*Crc*	This work
ΔCyoB	*P. putida* KT2440Δ*CyoB*	This work
ΔCrcΔCyoB	*P. putida* KT2440Δ*Crc*Δ*CyoB*	This work
ΔEstBCPZ	*P. putida* KT2440Δ*EstBCPZ*	This work
ΔEst12/K	*P. putida* KT2440Δ*Est12/K*	This work
ΔEstBCPZ12/K	*P. putida* KT2440Δ*EstBCPZ*Δ*Est12/K*	This work
* P. putida* ΔBOX	*P. putida* KT2440Δ*PP_2047-48*Δ*PP_2136-37*Δ*PP_2214-18*	This work
P-L	*P. putida* KT2440 harboring pCOM_*alkL*	This work
P-BGTL	*P. putida* KT2440 harboring pBTL10	This work
P-BGTLA	*P. putida* KT2440 harboring pBGTL-Atf1	This work
ΔCrc-BGTL	*P. putida* KT2440ΔCrc harboring pBTL10	This work
ΔCyoB-BGTL	*P. putida* KT2440ΔCyoB harboring pBTL10	This work
ΔCrcΔCyoB- BGTL	*P. putida* KT2440ΔCrcΔCyoB harboring pBTL10	This work
ΔCrc-BGTLA	*P. putida* KT2440ΔCrc harboring pBGTL-Atf1	This work
ΔCyoB-BGTLA	*P. putida* KT2440ΔCyoB harboring pBGTL-Atf1	This work
ΔCrc-BGTLAS	*P. putida* KT2440ΔCrc harboring pBGTL-Atf1, and pSEVAb628-alkS	This work
ΔCyoB-BGTLAS	*P. putida* KT2440ΔCyoB harboring pBGTL-Atf1, and pSEVAb628-alkS	This work
P-B^*^G^*^TLA^*^	*P. putida* KT2440 harboring pSEVAb658-B^*^G^*^TLA^*^	This work
ΔEst12/K- B^*^G^*^TLA^*^	*P. putida* KT2440ΔEst12/K harboring pSEVAb658-B^*^G^*^TLA^*^	This work
*E. coli* blank	*E. coli* NEBT7 harboring pET26b	This work
* E. coli*-EstX	*E. coli* NEBT7 harboring pET26b-EstX	This work

*HRs* = homologous regions

*Represents removal of Crc/Hfq mRNA attachment sites in the gene

**Table 2:** Putative esterases found in *P. putida* KT2440

Esterase name	Protein ID	Accession No.	Locus tag	Gene size (bp)
Est1	AAN65995.1	Q88QX0	PP_0364	732
EstB	AAN66920.1	Q88NB6	PP_1296	657
EstC	AAN66752.1	Q88NS7	PP_1127	1146
EstP	AAN66048.1	Q88QS0	PP_0418	1890
Est5	AAN67115.1	Q88MS5	PP_1493	1014
Est6	AAN69353.1	Q88GG5	PP_3759	576
EstZ	AAN69799.1	Q88F80	PP_4218	957
Est8	AAN69916.1	Q88EW5	PP_4337	1113
Est9	AAN70088.2	Q88EF1	PP_4514	2187
Est10	AAN70483.2	Q88DB1	PP_4916	609
Est11	AAN67697.1	Q88L52	PP_2083	1029
Est12/K	AAN69406.1	Q88GB2	PP_3812	1020
Est13	AAN70423.1	Q88DH1	PP_4854	891
Est14	AAN66966.1	Q88N71	PP_1343	912
Est15	AAN69866.1	Q88F13	PP_4286	927
Est16	AAN68665.1	Q88IE2	PP_3057	741

### Cultivation and gene expression

Strains harboring plasmids pCOM_alkL, pBTL10 or pBGTL-Atf1 were inoculated in LB overnight. The overnight culture (1% v/v) was used to inoculate M9 medium and cultured overnight again. OD_600_ was always determined spectrophotometrically with 4 mm cuvette. The day after, this overnight culture was used to inoculate 50 mL of M9 medium at an initial OD_600_ of 0.25, induced by 0.025% v/v dicyclopropylketone (DCPK) for gene expression. After 5-h induction at 30 °C and 250 rpm agitation in a Kuhner shaker incubator, cells were harvested by centrifugation at 4200 × *g* for 10 min. Cell pellets were suspended at 1 g_cdw_/L in resting cell buffer, containing 50 mM KPi pH 7.4, 2 mM MgSO_4_, and 1% v/v glucose. Strains harboring pSEVAb plasmids were cultivated under the same conditions as described above [46, 47], except for induction with 1 mM 3-methylbenzoate.

### Ester hydrolase screening and strain construction for tests

For the ester hydrolase screening, two main databases, NCBI and the Carbohydrate-Active enZYmes [48, 49], were used to search putative hydrolase-like esterase or lipase in *P. putida* KT2440. Each putative ester hydrolase gene (*EstX*) with a Strep-tag II at the C-terminal end was inserted into a pET26b plasmid to generate pET26b-EstX by HiFi assembly. All primers for these constructions are listed in Table S1. *E. coli* NEBT7 was transformed with the pET26b-EstX to give rise to *E. coli*-EstX for ester hydrolysis tests.

### Resting-cell hydrolysis assay

*E. coli*-EstX was inoculated in LB medium and grown overnight. An aliquot of the overnight culture (300 μL) was inoculated in 30 mL M9^E^ medium and grown overnight. This culture was used to inoculate 50 mL M9 medium at an initial OD_600_ of 0.15. After 5 h of cultivation (OD_600_ = 0.6–0.8), 0.1 mM isopropyl β-D-1-thiogalactopyranoside (IPTG) was added to induce ester hydrolase gene expression at 25 °C, at 250 rpm agitation in a Kuhner incubator for 16 h. Once induction finished, the cells were harvested by centrifugation at 4200 × g.

Cell pellets were suspended in the resting-cell buffer to a density of 1 g_cdw_/L. Aliquots of 1 mL resting cells were taken for each reaction in a Pyrex tube. Alkyl (C6-C10) acetate and diacetoxyhexane (20 mM) were added to each tube, and then the tube was cultivated for 20 h at 30 °C, 250 rpm. All reactions were performed in triplicate. Afterwards, 1% v/v of phosphoric acid was immediately added to stop reactions and all tubes were placed on ice. Samples were extracted by diethyl ether with 0.2 mM dodecane as an internal standard for GC analysis.

### Growth test on hexyl acetate and diacetoxyhexane medium

*P. putida* KT2440 was cultivated in LB overnight. The overnight culture (1% v/v) was used to inoculate 50 mL M9 medium in a 250-mL shake flask and cultivated overnight again. The day after, this overnight culture was used to inoculate 10 mL M9 medium in 50-mL tubes (10 mM hexyl acetate or diacetoxyhexane as the only carbon source) with an initial OD_600_ of 0.15. Three tubes were taken out every 24 h for optical density and substrate concentration assays. Samples for GC analysis were prepared as mentioned above.

### HPLC analysis

HPLC analysis was conducted with an Agilent 1260 Infinity UPLC with a 30 cm Rezex ROA column (Phenomenex), run at a flowrate of 0.8 mL/min with 5 mM H_2_SO_4_ as mobile phase and column temperature of at 25 °C. Quantitative analysis of metabolites was performed using a refractive index detector (RID).

### GC analysis

GC analysis was carried out on an Agilent 7890A gas chromatograph equipped with a flame ionization detector (FID) using the HP-5 column (30 m × 30 μm × 0.25 μm). 1 µL sample was injected in splitless mode, using the following temperature program: 50 °C for 3 min, 15 °C/min increase to 180 °C, 7 °C/min increase to 230 °C, 30 °C/min increase to 350 °C, which was held for 3 min. Quantification was done by using available standards. If standards were not commercially available, quantification was done on the basis of structurally related compounds with similar numbers of carbon/hydrogen atoms. For example, mono-hexyl adipate was quantified based on mono-ethyl sebacate.

## Supplementary Information

**Additional file 1: Table S1**. Primers used in this study.

## Data Availability

All data generated or analysed during this study are included in this published article and its additional information files.

## Abbreviations

Mcl-diols: Medium-chain-length α,ω-diols
CCR: Carbon catabolite repression
AlkS: Activator of alkane degradation pathway
Crc: Global regulator of carbon catabolite repression
Cyo: Cytochrome o ubiquinol oxidase
B: AlkB, Alkane monooxygenase
F: AlkF, rubredoxin which is indispensable for AlkB activity
G: AlkG, rubredoxin
L: AlkL, outer membrane protein
T: AlkT, rubredoxin reductase
S: AlkS, activator of alkane degradation pathway
PalkS2: Promoter of AlkS with efficient transcription in presence of alkanes
PalkS1: Promoter of AlkS in absence of alkanes
PalkB: Promoter of AlkB
CyoB: The key gene of cytochrome o ubiquinol oxidase
Atf1: Alcohol acetyltransferase
EST: Esterases
ADH: Alcohol dehydrogenase
ALDH: Aldehyde dehydrogenase
ALR: Aldehyde reductase
CAR: Carboxylic acid reductase
PPT: Phosphopantetheinyl transferase
6HHA: 6-Hydroxy hexyl acetate
DAH: 1,6-Diacetoxyhexane
Glc: Glucose
Cit: Citrate
KO: Knockout
BOX: Beta-oxidation
*FadA*: Acetyl-CoA acetyltransferase
*FadB*: 3-Hydroxyacyl-CoA dehydrogenase/enoyl-CoA hydratase
*FadE*: Acyl-CoA dehydrogenase
PES: Polyethersulfone
Km: Kanamycin
Gm: Gentamicin
DCPK: Dicyclopropylketone
*EstX*: Putative ester hydrolase gene
IPTG: Isopropyl β-D-1-thiogalactopyranoside
RID: Refractive index detector
FID: Flame ionization detector
