# Supplementary material for: When metabolic prowess is too much of a good thing: how carbon catabolite repression and metabolic versatility impede production of esterified α,ω-diols in Pseudomonas putida KT2440
Source: Biotechnol Biofuels. 2021 Nov 20;14:218. doi: 10.1186/s13068-021-02066-x (PMC8606055; doi:10.1186/s13068-021-02066-x)
Supplement: Supplementary file 1 — Additional file 1: Table S1. Primers used in this study. [file 13068_2021_2066_MOESM1_ESM.docx]

**When metabolic prowess is too much of a good thing: How carbon catabolite repression and metabolic versatility impede production of esterified α,ω-diols in *Pseudomonas putida* KT2440**

Chunzhe Lu^a^, Christos Batianis^b^, Edward Ofori Akwafo^a^, , Rene H. Wijffels^a,d^, Vitor A.P. Martins dos Santos^a,b,c^, Ruud A. Weusthuis^a^

a Bioprocess Engineering, Wageningen University and Research, Wageningen, The Netherlands

b Laboratory of Systems and Synthetic Biology, Wageningen University and Research, Wageningen, The Netherlands

c Lifeglimmer GmbH, Berlin, Germany

d Faculty of Biosciences and Aquaculture, Nord University, Bodø, Norway

**Plasmids construction**

The plasmid pSEVA65-alkS was constructed by HIFI-assembly. AlkS gene and pSEVA65 backbone including XylS/Pm were amplified by primers for pSEVA65-AlkS construction in Table S1. pSEVA65-BGTL-Atf1 was done by HIFI-assembly too. Primers for pSEVA65-AlkBGTL-Atf1 construction are listed in Table S1. 65-backbone Fwr/65 backbone Rvs2 was used to amplify pSEVA65 backbone including XylS/Pm. alkBG Fwr/alkFG Rvs was used to amplify alkBF genes. alkG Fwr/alkL Rvs was designed for gene amplification of alkG and alkL. Atf1 Fwr/Atf1 Rvs and AlkT Fwr/AlkT Rvs were used to amplify the fragments of Atf1 and AlkT genes, respectively. Afterward, all fragments (pSEVA65 backbone, alkBF, alkGL, Atf1, and AlkT) were assembled according to HIFI-assembly instruction.

The empty plasmid pET26b was used as template to amplify pET26b backbone by primers Vector NdeI Fwr/ Vector XhoI Rvs. All esterases (est1, B, C, P, 5, 6, Z, 8, 9, 10, 11, 12, 13, 14, 15, 16) was amplified according to primers listed in Table S1. Each fragment was assembled with of esterase pET26b backbone to generate pET26b-EstX, in which X represents one of esterase.

The plasmids pGNW-Δcrc and pGNW-ΔcyoB were constructed for *crc* and *cyoB* deletion. pGNW Fwr/pGNW Rvs was used to amplify pGNW backbone. TS1 Crc Fwr/TS1 Crc Rvs and TS2 Crc Fwr/TS2 Crc Rvs were designed to amplify 500bp homologous arm upstream (TS1) and downstream (TS2) of *crc* and *cyoB*. TS1-Crc and TS2-Crc was assembled with pGNW backbone by Golden Gate, generating pGNW-Δcrc. TS1-CyoB and TS2-CyoB was assembled with pGNW backbone by Golden Gate, generating pGNW-ΔcyoB.

The plasmids pGNW-ΔPP2047-48, pGNW-ΔPP2136-37, and pGNW-ΔPP2214-17 were constructed to interrupt β-oxidation. Primers TS1 2047-48 Fwr/TS1 2047-48 Rvs, and TS2 2047-48 Fwr/TS2 2047-48 Rvs were used to amplify 500bp homologous arm upstream and downstream of the gene PP_2047-48. Primers TS1 2136-37 Fwr/TS1 2136-37 Rvs, and TS2 2136-37 Fwr/TS2 2136-37 Rvs were used to amplify 500bp homologous arm upstream and downstream of the gene PP_2136-37. Primers TS1 2214-17 Fwr/TS1 2214-17 Rvs, and TS2 2214-17 Fwr/TS2 2214-17 Rvs were used to amplify 500bp homologous arm upstream and downstream of the gene PP_2214-17. TS1 and TS2 of each genes fragment needed to be deleted were assembled with pGNW backbone, giving rise to pGNW-ΔPP2047-48, pGNW-ΔPP2136-37, and pGNW-ΔPP2214-17.

The plasmid pGNW-ΔestX were constructed for *estX* deletion. Primers TS1 estX Fwr/TS1 estX Rvs, and TS2 estX Fwr/TS2 estX Rvs were used to amplify 500bp homologous arm upstream and downstream of the gene estX. TS1-estX and TS2-estX was assembled with pGNW backbone by Golden Gate, generating pGNW-ΔestX.

Table S1 Primers used in this study

| Primers | Sequences (5’-3’) |
| --- | --- |
| Primers for pET26b-*EstX* construction | |
| Vector NdeI Fwr | catatgtatatctccttcttaaagttaaacaaaattatttctagaggg |
| Vector XhoI Rvs | TGGAGCCACCCGCAGTTCGAAAAATAACTCGAGgctgctaacaaagcccgaaagg |
| 1-NdeI-Fwr | actttaagaaggagatatacatATGCGTAATCGACTTGTTCTATTGCCCG |
| 1-Strep-XhoI-Rvs | TTATTTTTCGAACTGCGGGTGGCTCCaGTCATCGCCACTCTCATGCAG |
| estB-NdeI-Fwr | actttaagaaggagatatacatatgaccaacccgctgattctcgaac |
| estB-Strep-XhoI-Rvs | TTATTTTTCGAACTGCGGGTGGCTCCAttgcagctgcttgctcagcca |
| estC-NdeI-Fwr | actttaagaaggagatatacatAtgcagatccagggtcactatgagc |
| estC-Strep-XhoI-Rvs | TTATTTTTCGAACTGCGGGTGGCTCCAaaggcaactggcaaggacgc |
| estP-NdeI-Fwr | actttaagaaggagatatacatatgcgaaaagccccgTTATTGCG |
| estP-Strep-XhoI-Rvs | TTATTTTTCGAACTGCGGGTGGCTCCAgaagtccacgctaacccccac |
| 5-NdeI-Fwr | actttaagaaggagatatacatATGAAGATCGCCATCGTCAATGACATGC |
| 5-Strep-XhoI-Rvs | TTATTTTTCGAACTGCGGGTGGCTCCaTTTTGCGAAAAATTCAGTCAAGCGC |
| 6-NdeI-Fwr | actttaagaaggagatatacatATGAGCGGCGTACGCGC |
| 6-Strep-XhoI-Rvs | TTATTTTTCGAACTGCGGGTGGCTCCaGCATGCGCTGTATTCCAGGGTC |
| estZ-NdeI-Fwr | actttaagaaggagatatacatatgtccctgaaccctgacctgg |
| estZ-Strep-XhoI-Rvs | TTATTTTTCGAACTGCGGGTGGCTCCAcagatgctgctgcagtgcctc |
| 8-NdeI-Fwr | actttaagaaggagatatacatATGGCAGTCAAGGTCCTGGTGG |
| 8-Strep-XhoI-Rvs | TTATTTTTCGAACTGCGGGTGGCTCCaGACGCACGCCTCCACC |
| 9-NdeI-Fwr | actttaagaaggagatatacatATGCGCCGTTTGCTGTTCTGTTTTC |
| 9-Strep-XhoI-Rvs | TTATTTTTCGAACTGCGGGTGGCTCCaGAAGGTGTGCCCAAGGTTCA |
| 10-NdeI-Fwr | actttaagaaggagatatacatATGTCGGGTTCCATCCTCTATATCCATGG |
| 10-Strep-XhoI-Rvs | TTATTTTTCGAACTGCGGGTGGCTCCaAAATACAGAAAAATCGAGCGCCGCATAC |
| 11-NdeI-Fwr | actttaagaaggagatatacatATGCAAAGCAGCAGCACTTTATTCCCC |
| 11-Strep-XhoI-Rvs | TTATTTTTCGAACTGCGGGTGGCTCCaGCTGGCAGGCACAGGTTC |
| 12-NdeI-Fwr | actttaagaaggagatatacatATGAACATTGTCCACAAAGCCCTCAC |
| 12-Strep-XhoI-Rvs | TTATTTTTCGAACTGCGGGTGGCTCCacTGCAGATGCACTTTAAGTTCGT |
| 13-NdeI-Fwr | actttaagaaggagatatacatATGGTCATGATGGCCGCTGTA |
| 13-Strep-XhoI-Rvs | TTATTTTTCGAACTGCGGGTGGCTCCacAAGCCCCGCCGCCTTG |
| 14-NdeI-Fwr | actttaagaaggagatatacatATGATTAAACAACGCACCCTGAAGAATACC |
| 14-Strep-XhoI-Rvs | TTATTTTTCGAACTGCGGGTGGCTCCacACGGCCGCAACAGGG |
| 15-NdeI-Fwr | actttaagaaggagatatacatATGAGCGCTGATTACCCTCGC |
| 15-Strep-XhoI-Rvs | TTATTTTTCGAACTGCGGGTGGCTCCaGGCGTTCTCATTCTTGTACGGGTG |
| 16-NdeI-Fwr | actttaagaaggagatatacatatgagtctcgaaactgaaattgcagg |
| 16-Strep-XhoI-Rvs | TTATTTTTCGAACTGCGGGTGGCTCCAGAGCTTGGTGATATTGGTGGTC |
| Sequence Fwr | ccaccatacccacgccgaaa |
| Sequence Rvs | gcccccgatttagagcttga |
| Primers for crc and cyoB deletion | |
| TS1 Crc Fwr | aGGTCTCtcccggatgatctgcatgacctcacgaat |
| TS1 Crc Rvs | aGGTCTCaCCTTaaaatggccccataaatctcgtgc |
| TS2 Crc Fwr | aGGTCTCgaaggccattggggctgcattg |
| TS2 Crc Rvs | aGGTCTCttcgaaacgccatgctcgctttggcg |
| g Check1 Fwr | gttcagcgcgatcagtacaccag |
| g Check1 Rvs | agccgaaaccgacctgaatgtgg |
| TS1 cyoB Fwr | aGGTCTCtcccgtcaacaagatcgtcttccc |
| TS1 cyoB Rvs | aGGTCTCaTTGActcttactcctctgcaccgg |
| TS2 cyoB Fwr | aGGTCTCgtcaatgtccagtcaagtaat |
| TS2 cyoB Rvs | aGGTCTCttcgatggtacatcatgattgccat |
| gCheck ext cyoB Fwr | gacccttatcgtccgctgga |
| gCheck ext cyoB Rvs | cggtgaatacgcagatccaga |
| Primers for β-oxidation deletion | |
| TS1 2047-48 Fwr | aGGTCTCtcccgaggtgcaccacgtcgatgc |
| TS1 2047-48 Rvs | aGGTCTCaTGCGgcgcaacgctcctgctttcttg |
| TS2 2047-48 Fwr | aGGTCTCgcgcacggcaaaccacacg |
| TS2 2047-48 Rvs | aGGTCTCttcgagtggtgacgatggcgatcgg |
| g Check1 2047-48 Fwr | atctgcagggtttcacggcg |
| g Check1 2047-48 Rvs | gtagcagcagtcaccgccg |
| TS1 2136-37 Fwr | aGGTCTCtcccgatccaggctggtcaatctgttctg |
| TS1 2136-37 Rvs | aGGTCTCaGCGAcaactgatctccacgatatggaag |
| TS2 2136-37 Fwr | aGGTCTCgtcgcgtagcgggacagcag |
| TS2 2136-37 Rvs | aGGTCTCttcgacgtactggttgcccaggtacttg |
| g Check1 2136-37 Fwr | CGGCCATAGAATCTCCTACGGG |
| g Check1 2136-37 Rvs | cttgccaggcaggatctcgtactt |
| TS1 2214-17 Fwr | aGGTCTCtcccgagctgggtatcaccaacctg |
| TS1 2214-17 Rvs | aGGTCTCaACTGgagtactttcctttcagacgct |
| TS2 2214-17 Fwr | aGGTCTCgcagtggaagcaaattcgca |
| TS2 2214-17 Rvs | aGGTCTCttcgaaaacgcaaaaagccccagggat |
| FadBAEx_Int_F | GACCTCAAGAGCCTGACTGC |
| FadBAEx_Int_R | GCCGTGGATATTGACCTTGT |
| FadBAEx_Ext_F | TTGGGCTTACGGCTTGTATT |
| FadBAEx_Ext_R | ACACCCGACCCTATCATCAC |
| Primers for pSEVA65-AlkS construction | |
| 65 backbone Fwr | GACTCCTGTTGATAGATCC |
| 65 backbone Rvs | ATGTTCATGACTCCCCTAG |
| alkS Fwr | CCTAGGGGAGTCATGAACATatgaaaataataataaataatgatttccc |
| alkS Rvs | ATTACTGGATCTATCAACAGGAGTCttagataattccttgacgc |
| Primers for pSEVA65-AlkBGTL-Atf1 construction | |
| 65-backbone Fwr | GACTCCTGTTGATAGATCC |
| 65 backbone Rvs2 | ctagtaTTTCTCCTCTTTctctagtaTAGTaCTAGTAattattgtttctgttgcataaa |
| alkBG Fwr | tactagagAAAGAGGAGAAAtactagatgcttgagaaacacaga |
| alkFG Rvs | CTTTctctagtagcggcaaatttgcgttatt |
| alkG Fwr | atttgccgctactagagAAAGAGGAGAAAtactagatggctagctataaatgcc |
| alkL Rvs | tagtattagaaaacatatgacgcacc |
| Atf1 Fwr | tcatatgttttctaatactagagAAAGAGGAGAAAtactagatgaacgaaatcgacgaaaag |
| Atf1 Rvs | agtattacggacccagcagcagtg |
| alkT Fwr | gctgctgggtccgtaatactagagAAAGAGGAGAAAtactagATGGCGATTGTCGTCGTC |
| alkT Rvs | GGATCTATCAACAGGAGTCTTAGTCGGGGAGCTTGATGG |
| Primers for esterases deletion | |
| TS1 EstB Fwr | aGGTCTCtcccgGCCCTGCGCATCAGGT |
| TS1 EstB Rvs | aGGTCTCaCTGCaGACTGCTCCGAAAGTGTGCG |
| TS2 EstB Fwr | aGGTCTCgGCAGCCTCGCTTGTA |
| TS2 EstB Rvs | aGGTCTCttcgaGGCCGATGGCGTCCTG |
| g check EstB Fwr | tgagctggctgaacttg |
| g check EstB Rvs | catgtagcgctctttagg |
| TS1 EstC Fwr | aGGTCTCtcccgCGGCTACGACTTTGCC |
| TS1 EstC Rvs | aGGTCTCaCCGACCGCTGCTCTCCGTAC |
| TS2 EstC Fwr | aGGTCTCgTCGGGTAATGCGGGTATTGC |
| TS2 EstC Rvs | aGGTCTCttcgaTCAGGATTGTACGAGC |
| g check EstC Fwr | cctgggtggccgctatg |
| g check EstC Rvs | ctgtcggcatggcccagt |
| TS1 EstP Fwr | aGGTCTCtcccgACGTACAACGTCGCCG |
| TS1 EstP Rvs | aGGTCTCaCAATAGACCTATCCTGTGTTGATCGT |
| TS2 EstP Fwr | aGGTCTCaATTGCTGGGGGCCGC |
| TS2 EstP Rvs | aGGTCTCttcgaACATTGTTTCCAACGT |
| g check EstP Fwr | ggttgttaaggctggcga |
| g check EstP Rvs | ctgatcgatctgggccg |
| TS1 EstP Fwr | aGGTCTCtcccgACGTACAACGTCGCCG |
| TS1 EstZ Rvs | aGGTCTCaTCCCCGTTCGATTCTCCAGAGGGG |
| TS2 EstZ Fwr | aGGTCTCaGGGAGCCTTTCAGGC |
| TS2 EstZ Rvs | aGGTCTCttcgaACCCCCAACTGGCAAG |
| g check EstZ Fwr | CTTGGCGTCACTGATGG |
| g check EstZ Rvs | GCAGAAGCTTCTAACCAGG |
